# Effects of shell-integrated Sudan Black dye on the acoustic activity and ultrasound imaging properties of lipid-shelled nanoscale ultrasound contrast agents

**DOI:** 10.1117/1.JBO.27.1.016501

**Published:** 2022-01-21

**Authors:** Dana Wegierak, Grace Fishbein, Eric Abenojar, Al De Leon, Jinle Zhu, Yanjie Wang, Charlotte Ferworn, Agata A. Exner, Michael C. Kolios

**Affiliations:** a Ryerson University, Faculty of Science, Department of Physics, Toronto, Canada; b Case Western Reserve University, Department of Biomedical Engineering, Cleveland, United States; c Case Western Reserve University, Department of Radiology, Cleveland, United States

**Keywords:** nanobubbles, contrast agents, photoacoustic imaging, ultrasound imaging, multimodal imaging, rectified diffusion

## Abstract

**Significance:**

An effective contrast agent for concurrent multimodal photoacoustic (PA) and ultrasound (US) imaging must have both high optical absorption and high echogenicity. Integrating a highly absorbing dye into the lipid shell of gas core nanobubbles (NBs) adds PA contrast to existing US contrast agents but may impact agent ultrasonic response.

**Aim:**

We report on the development and ultrasonic characterization of lipid-shell stabilized $C_{3}F_{8}$ NBs with integrated Sudan Black (SB) B dye in the shell as dual-modal PA-US contrast agents.

**Approach:**

Perfluoropropane NBs stabilized with a lipid shell including increasing concentrations of SB B dye were formulated by amalgamation (SBNBs). Physical properties of SBNBs were characterized using resonant mass measurement, transmission electron microscopy and pendant drop tensiometry. Concentrated bubble solutions were imaged for 8 min to assess signal decay. Diluted bubble solutions were stimulated by a focused transducer to determine the response of individual NBs to long cycle (30 cycle) US. For assessment of simultaneous multimodal contrast, bulk populations of SBNBs were imaged using a PA and US imaging platform.

**Results:**

We produced high agent yield ($∼10^{11}$) with a mean diameter of ∼200 to 300 nm depending on SB loading. A 40% decrease in bubble yield was measured for solutions with 0.3 and 0.4  mg/ml SB. The addition of SB to the shell did not substantially affect NB size despite an increase in surface tension by up to 8  mN/m. The bubble decay rate increased after prolonged exposure (8 min) by dyed bubbles in comparison to their undyed counterparts (2.5-fold). SB in bubble shells increased gas exchange across the shell for long cycle US. PA imaging of these agents showed an increase in power (up to 10 dB) with increasing dye.

**Conclusions:**

We added PA contrast function to NBs. The addition of SB increased gas exchange across the NB shell. This has important implications in their use as multimodal agents.

## 1 Introduction

Microvascular morphology is a hallmark of many diseases, including cancer.^1^ Medical imaging modalities, such as photoacoustic (PA) and ultrasound (US) imaging, can effectively assess the morphology of microvasculature. In PA imaging, ultrasonic pressure waves are generated from the thermoelastic expansion of tissue chromophores after laser absorption. Naturally present in the human body, hemoglobin in red blood cells is a strong light absorber and therefore generates a relatively high PA signal.^1^ On the other hand, US imaging utilizes acoustic waves that are generated by a transducer, transmitted through tissue, and reflected off boundaries of tissues that exhibit differences in acoustic impedance. For instance, in Doppler US, erythrocytes scatter the sound, giving rise to US echoes. The morphological and flow information provided by US imaging, paired with the functional information provided by PA imaging can be used to evaluate tumor morphology while tracking tumor metabolism.^2^ As such, PA-US multimodal imaging is a promising tool for early tumor detection and diagnosis.

Despite the potential of PA-US multimodal imaging, there are cases in which both modalities fall short. US is limited by relatively small differences in acoustic impedance in the body and weak scattering from red blood cells.^3^ Additionally, the number of photons available for PA imaging significantly decreases with depth, which results in lower amplitude acoustic waves that may not be sensed by the transducer.^3^ There is a need to: (1) extend the imaging depth of PA imaging and (2) enhance US contrast to improve cancer diagnosis. Exogenous contrast agents have been used with both modalities to meet these needs. US contrast agents typically employ a gas vesicle to achieve an increase in acoustic impedance mismatch with surrounding tissue, preferably with a size near its resonance frequency for the generation of nonlinear bubble response, including harmonics. Microbubbles (MBs) with an average of 2 to 3  μm in diameter and typically stabilized with a protein, polymer, or lipid shell are now the dominant agent used for US enhancement.^4^^–^^7^ Exogenous PA contrast agents include gold nanoparticles, indocyanine green dye (ICG) dye, and numerous other absorbers.^8^^,^^9^ Synthesizing multimodal contrast agents that can harness the benefits of both acoustic and PA imaging is an important step toward quantitative multimodal imaging that seamlessly integrates PA with US.

To date, several contrast agents have been investigated for combined simultaneous PA-US imaging. A successful agent to be used for both US and PA imaging must exhibit two key properties in comparison to surrounding tissue: (1) high acoustic scattering and (2) high optical absorption. One solution is the use of previously approved US contrast agents (MBs FDA approved and NBs) coated with optical absorbers such as nanoparticles and dyes.^3^^,^^10^^,^^11^ Dual-mode acoustic/PA contrast agents have also been investigated in various pre-clinical applications.^12^ Several groups have demonstrated use *in vivo* imaging.^13^^–^^15^ For example, Das et al.^16^ generated nitrogen microbubbles for dual modal US/PA *in vivo* animal imaging of the urinary bladder of rats. Loaded PA nanodroplets, which can be forced to undergo a phase change when mediated by an external optical energy source, have been suggested as another potential solution. Due to the controllable phase change, PA nanodroplets function as activatable contrast agents.^17^^,^^18^ However, narrow absorption spectra limit the wavelength at which agent activation can occur thereby limiting range of systems with which the same agent can be used. Additionally, because the agents are not FDA approved, translation to clinic may experience significant delays. Beyond imaging, dual-mode agents have been applied to deliver therapeutic agents to the disease site to accomplish simultaneous imaging and therapy, or theranostics.^11^^,^^19^^,^^20^ For instance, recently our group synthesized perfluorohexane nanoemulsions which offered simultaneous US and PA contrast enhancement. These agents were shown as effective for treating MCF-7 cancer cells and monitoring tumor regression through imaging.^21^

Despite the progress that has been made, MB-based agents have several limitations. In tumors, the aggressive growth of the neoplastic cell population and associated overexpression of pro-angiogenic factors leads to the development of disorganized blood vessel networks that are fundamentally different from the normal vasculature (i.e., leaky).^22^^–^^24^ In the context of vascular imaging and cancer detection, MBs are restricted to the microvasculature, and therefore are less effective as theranostic agents for less vascularized tissue. Even in the case of a leaky vasculature, in which cellular gaps in vessel walls of tumors are enlarged through the enhanced permeability and retention effect,^24^ MBs remain intravascular contrast agents. To extravasate, bubbles need to be less than 800 nm in diameter.^25^ NBs (∼100 to 500 nm diameter) coated with optical absorbers can be used as PA-US multimodal agents with theranostic potential. Additionally, NBs, like their larger counterparts, are capable of the nonlinear responses to incident US^26^ thought to be the main driver for effective US contrast enhancement. Combining NBs with a PA contrast agent thus provides an attractive platform for new diagnostic and theranostics approaches. Accordingly, in this work, we examined the use of Sudan Black (SB) B dye to transform NB US contrast agents into multimodal agents for combined PA-US imaging.

**Fig. 1:**
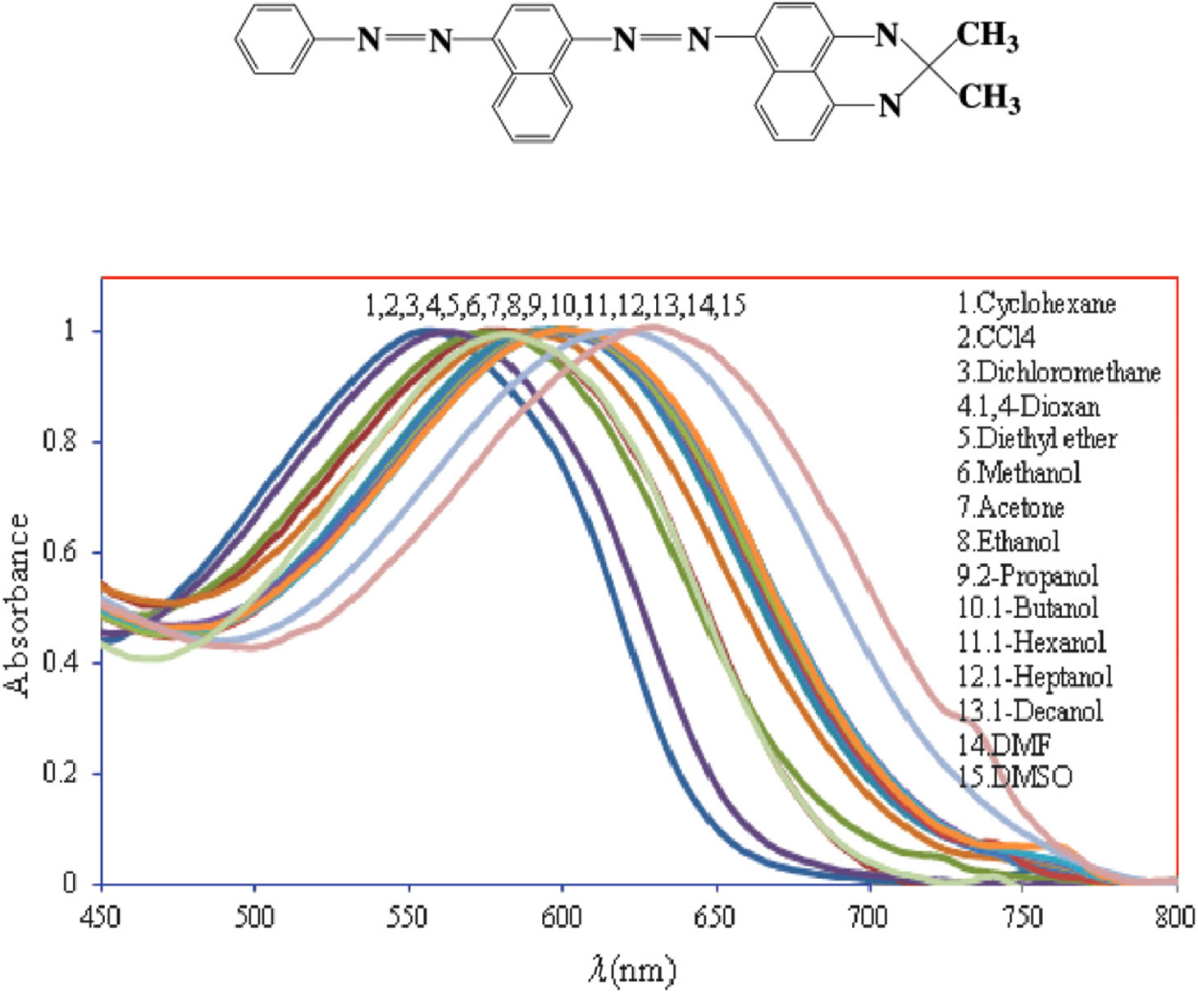
(a) Two-dimensional chemical structure of SB B and (b) optical absorption spectrum of SB B dye in different solvents^13^ (reproduced with permission).

SB [Fig. 1(a)^27^] is a fat-soluble dye that has been used for the sensitive and specific staining of phospholipids and intracellular lipids and can therefore be easily integrated into the lipid shell of an encapsulated NB. The optical absorption of SB dye has also been studied and established by previous groups.^27^ To date, few clinical studies using SB have demonstrated its feasibility for *in vivo* use. However, SB B has been used *in vivo* for the comparison of techniques for obturating oval-shaped root canals^28^ and to evaluate chyle leakage.^29^^,^^30^ In other applications, SB has mainly been applied in *ex vivo* staining protocols due to its lipophilic nature.^31^^,^^32^ There appears to be inadequate evidence to specify its biocompatibility at this time.^30^

We recently reported on the first feasibility test of the formulation of SB lipid-shelled NBs.^33^ However the physical or acoustic characterization of these NB agents has not previously been studied. Dye integration in the NB shell can be expected to have an impact on bubble dynamics and bubble stability under US stimulation due to the sensitive dependence of bubble oscillations on the NB shell parameters.^34^^–^^36^ Currently, FDA approved dyes for *in vivo* imaging includes methylene blue and indocyanine green. Methylene blue has a molecular weight similar to SB B (methylene blue–319.9 daltons; SB–456.5 daltons) and hydrophobic analogues of methylene blue are also available. Meanwhile, indocyanine green has a much larger molecular weight (775 daltons) than SB B but similar overall structure with three separate ring domains which lend to the optical properties of the molecule.^37^ As such, currently FDA approved dyes may have similar effects to those with SB on a lipid shelled gas vesicle intended for multimodal PA and US use.

Here, we present a study of the acoustic response of the lipid shelled NB US contrast agents after integration of SB dye. NB formulations with varying SB B loading were produced, and physical properties of the agents were characterized using transmission electron microscopy (TEM) and resonant mass measurement (RMM). The bulk shell-material surface tension was assessed through pendant drop tensiometry. The acoustic response of gas-core agents is sensitive to US exposure parameters such as pressure, frequency, and pulse length. In this work, the responses of bubble populations and single bubbles were assessed using systems that offered different functional exposure capabilities, including simultaneous US and PA multimodal contrast imaging.

## 2 Methods

### 2.1 Lipid Materials

The lipids 1,2-dibehenoyl-sn-glycero-3-phosphocholine, 1,2-dipalmitoyl-*sn*-glycero-3-phosphate, and 1,2-dipalmitoyl-*sn*-glycero-3-phosphoethanolamine were obtained from Corden Pharma (Switzerland), and 1,2-distearoyl-*sn*-glycero-3-phosphoethanolamine-N-[methoxy(poly(ethylene glycol))-2000] (ammonium salt) was obtained from Laysan Lipids (Arab, Alabama). All materials were used as received. When not in use, lipids were stored at −20°C.

### 2.2 Lipid Solution Preparation

NB samples were produced in-house. The base protocol for the production of shell-stabilized NBs used for the current work was reported elsewhere.^35^ The protocol with the addition of SB differs minimally from the published work. For the synthesis of the shell solution the lipids (see Supplementary Material) were dissolved in glycerol (Acros Organics, Morris Plains, New Jersey) under heat at 80°C for 30 min. SB dye (Sigma Aldrich, Cleveland, Ohio) was measured in volumes of 0, 1, 2, 3, 4, and 5 mg and added to dissolved volumes for lipids measured to produce a 10-ml batch. Once SB was fully dissolved in lipids under heat without visible precipitates, phosphate buffered saline (PBS) and glycerol was added to the solution and sonicated for a final 10 ml yield of SB + lipid solution. The 10 ml yield was aliquoted into ten 1-ml aliquots in 1.5-ml vials and sealed by a rubber cap. The final SB concentrations in the vials were 0, 0.1, 0.2, 0.3, 0.4, and 0.5 mg SB per 1-ml lipid solution.

### 2.3 NB Preparation

To prepare the vials for activation, air was removed from each vial manually by needle and syringe. 10 ml of octafluoropropane (C3F8) (Synquest Laboratories, Alachua, Florida) was then injected into the vial while a second needle was used for venting during the process. The NBs were formed by mechanical agitation for 45 s using a Vialmix mechanical shaker [Bristol Myers Squibb (BMS), New York City, New York]. The vial was then inverted, and differential centrifugation was used to separate the bubbles by size.^35^ After centrifugation, the vial was kept inverted during the sample draw to limit mixing of activated bubbles and control for bubble size distribution. The penetration depth of the 18 G needle into the vial was limited to 5 mm for a consistent draw. 300  μL of activated bubbles were drawn from the bottom of each vial and was used as activated, stock bubble solution. To further narrow the agent size distribution, the resulting isolated NB solutions were filtered. Here, activated stock bubble solution was diluted in PBS in a 1:9 dilution and passed through a single-use polyethersulfone filter unit (FroggaBio, Toronto, Ontario, Canada) with 450-nm pore size. The syringe was depressed by hand at a rate of one droplet per 5 s and the refuse was collected for immediate use.

### 2.4 Measurement of Bubble Concentration and Size

**Fig. 2:**
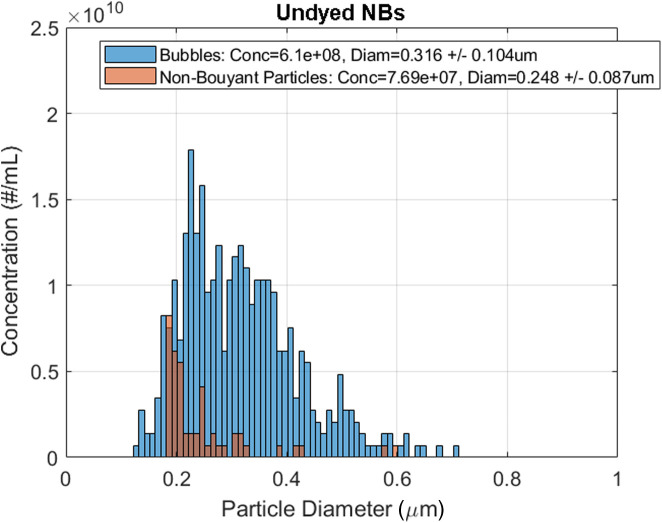
Sample size distribution of NBs and nonbuoyant particles prepared from lipid solution without SB before pore filtration as measured using the Archimedes RMM system.

A 1:500 dilution with PBS (pH 7.4) of activated bubbles from stock bubble solution was prepared for each sample type in PBS and sized using the Archimedes RMM system (Malvern Panalytical, Inc). RMM has been established as a technique for counting and sizing of suspended buoyant and nonbuoyant particles^36^ including NBs.^38^ Using RMM, buoyant particle concentration and diameter were determined. A nanosensor that provides measurements from 100 nm to 2  μm was used to characterize the NBs. The nanosensor was previously calibrated with National Institute of Standards and Technology (NIST) traceable 565-nm polystyrene bead standards, (ThermoFisher 4010S, Waltham, Massachusetts). About 500 particles were measured for each trial performed (n=3). The sensor and microfluidic tubing were cleaned with deionized (DDI) water in between each run. Data were exported from the Archimedes software (version 1.2) and analyzed for positive and negative counts, which corresponded to buoyant (bubble) and nonbuoyant particles, respectively. A density of $0.008\;{g\cdot \mathrm{ml}}^{-1}$ for positively buoyant particles (density of 0.008  g/cc) and $1.3\;{g\cdot \mathrm{ml}}^{-1}$ for negatively buoyant particles (density of 1.34  g/cc) were used to convert the measured mass to a particle diameter. A sample distribution for the Archimedes system is shown in Fig. 2. Filtered bubbles were prepared in 1 in 10 dilutions in PBS due to lower bubble yield from the filtration process. This process was repeated three times for each sample type.

### 2.5 Transmission Electron Microscopy (TEM)

TEM images of the bubbles were obtained using a FEI Tecnai G2 Spirit BioTWIN TEM operated at 120 kV based on a previously reported method.^39^^,^^40^ A dilute suspension of the sample (10  μL) was placed upside down on a 400 mesh Formvar-coated copper grid. The sample was then stained with 2% uranyl acetate, after which the sample was allowed to dry for 30 min.

### 2.6 Surface Tension

A CAM 200 optical goniometer by KSV Instrument, Ltd. with a pendant drop tensiometry setup was used to measure the surface tension of the various sample types at a lipid to air interface. A flat tipped needle (0.48-mm O.D.) was used. To obtain the most accurate results,^41^ samples were extruded from the needle to hold the largest possible, stable droplet. Water was used for calibration of the system. Measurements of water and lipid droplets were collected at 22.3 +/−0.3°C.

### 2.7 Acoustic Stability

**Fig. 3:**
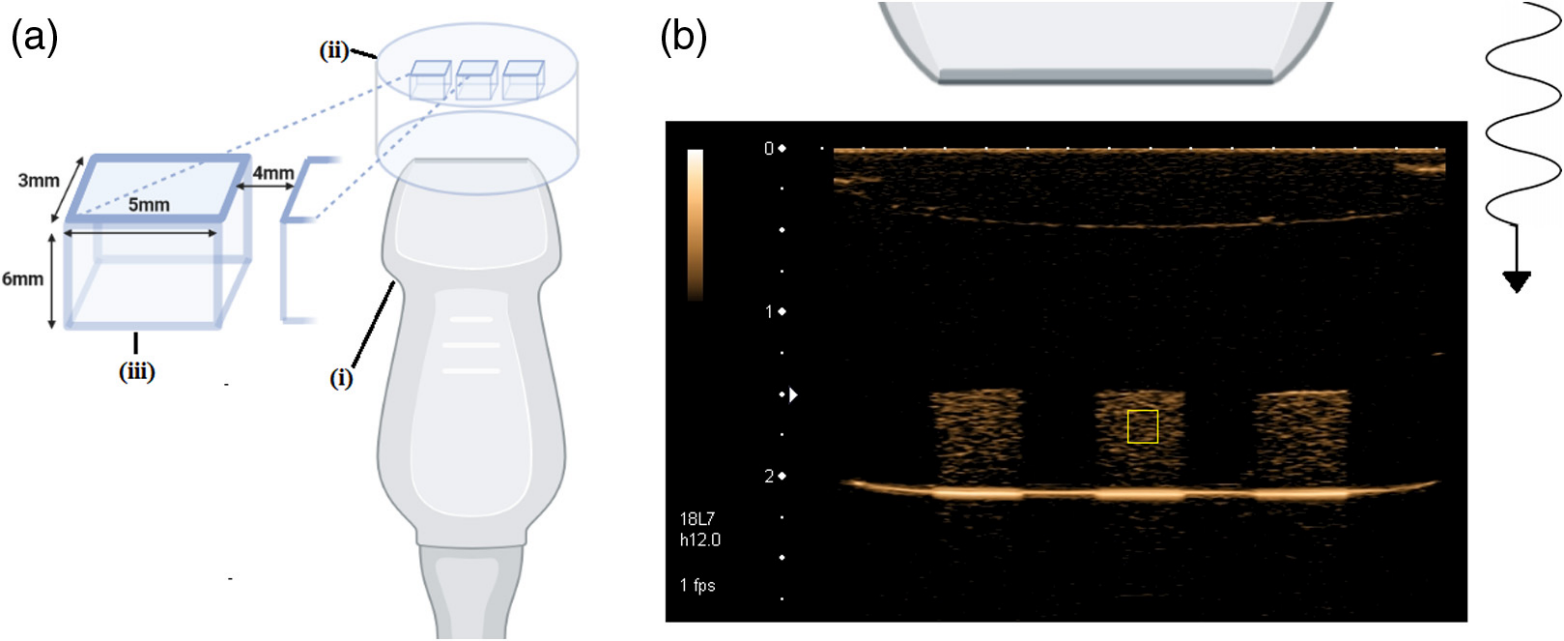
(a) Inverse bubble stability setup featuring a linear transducer (Toshiba, Tochigi-Ken, Japan, 6-MHz transmit, 12-MHz receive, 240 kPa peak negative pressure, M.I, 0.1), and 1% agarose mold with three narrow channels (3×5×6  mm) separated 4 mm apart; (b) US images from the inverted setup; Image of 1 in 100 diluted 0.5  mg/ml SB B NBs in PBS; top of image corresponds to the top of the transducer, bottom of image corresponds to air-exposed top of channel; sample ROI indicated by yellow box; figure created with BioRender.com.

To characterize changes in bubble echogenicity over time under constant insonation and in a more clinically relevant setting, grayscale intensity changes generated by the SBNBs were measured in vitro using a linear transducer (Toshiba, Tochigi-Ken, Japan) and a clinical US scanner (Toshiba Aplio) at 6-MHz transmit frequency, 12 MHz receiving frequency and a peak negative pressure of 240 kPa (mechanical index, 0.1). This data were collected using nonlinear contrast mode imaging. Phantoms were custom designed from agarose mold (1% agarose, $99\%H_{2}O$). Each phantom had three narrow channels (see Fig. 3). Bubbles were imaged using US for 8 consecutive minutes. The phantom channels were aligned to the center of the transducer element placed atop such that the transducer was in an inverted orientation.

Activated bubble solution was prepared at constant dilution of stock solution (1 in 100). Six images were acquired for each sample. A ROI was selected within each channel region. The average US power of all non-zero elements in a selected ROI was tracked with respect to time for the duration of the 8 min excitation. This data was averaged for all six measurements and normalized to data from a noise region within the image.

### 2.8 Single Bubble Ultrasound Scattering

The acoustic response of single Sudan Black nanobubble (SBNB) agents was studied using the Vevo 770 US system. The high frequency of the transducer paired with the adjustable pulse length was important for localization of signals from single agents. A focused RMV-710B transducer (f-number 2.1; aperture 7.14 mm; focal length 15 mm) with center frequency of 25 MHz and 100% bandwidth was used. This transducer has a mechanical scan head that oscillates laterally as it scans a fixed region of interest. Bubbles were stimulated with 30 cycle pulses at 25 MHz at varying pressures (0.2, 0.6, 1, 2, and 3 MPa). For each power setting, 25 data sets were collected. Each data set contained 100 RF lines where one RF line represents the measured backscatter data associated with the material along the axial path of the transducer.^42^^–^^44^ This experiment was repeated for each of three different vials of the 0, 0.2, and 0.4 mg SB samples three times each (nine total).

Stock bubble solutions were filtered by pore filtration to minimize size-dependent effects in SBNB stimulation. After filtration, the bubble concentration was determined and used to prepare a dilution with a consistent number of NBs per volume in DI for each measurement (∼2000  NBs/ml). The diluted bubble solution was gently stirred to disperse the bubbles. The transducer head was submerged into the beaker and held in place 3 cm below the surface of the water. A new dilution was prepared for each measurement to ensure high signal detection that was concentration independent. A sample image of the raw data from the system is available in the Supplementary Material.

Analysis of the RF data was done using MATLAB R2019b. RF data were converted from time to frequency domain to yield the power spectra of the data. The data was then subject to a set of criteria for identification as single bubble signals. RF lines lacking bubbles, truncated RF lines, or lines with signals from more than one bubble were removed. The remaining accepted signals were finally classified as either linear, nonlinear, or transient. A linear oscillation has a minimum and maximum radius that is consistent throughout the US excitation. Such signals were labeled p1. In some cases, linear signals occurred such that a steady increase or decrease in amplitude with cycle number was detected. Such signals were labeled as ${p1}_{\text{growth}}$ or ${p1}_{\text{diss}}$ respectively.

Nonlinear signals were identified through consistent periodicity, which was identified through patterns that occurred in the time domain signal as a function of cycle number (every two peaks; every three peaks; and every four peaks) and thus were labeled p2, p3, p4, p5, p6, or p7 depending on the pattern observed (see Supplementary Material).^35^^,^^44^ The most commonly occurring repetitive sequence in the RF line was used to classify the signal behavior. For some signals, no clear pattern in oscillation was visible, however the oscillations were nonlinear. These signals have been denoted $p_{x}$. To account for the low numbers of nonlinear signals detected overall, the nonlinear signals were all grouped together under the common classification, $p_{n}$

Destructive signals or cavitation signals were those such that the signal demonstrated a bubble that does not complete all 30 oscillations and power spectra with visibly broadband shapes in comparison to other signals.

Classified signals were examined to track the total number of signals that are classified into the five groups: (1) linear, (2) linear and increasing in amplitude (growth), (3) linear and decreasing in amplitude (dissolution), (4) nonlinear, and (5) cavitation signals.

### 2.9 Multimodal PA-US Imaging

PA and US multimodal imaging was done using the Vevo LAZR 2100 system with LZ250 transducer operating at 21 MHz central frequency (13-24 MHz bandwidth). The transducer is a 256-element linear array transducer coupled with a laser (ƛ=680–970  nm) system. The transducer and optical illumination share a common focus at 11-mm imaging depth.^45^ US and PA RF data were acquired simultaneously when acquiring PA images. US images were collected at 1% transducer power (peak negative pressure 0.922 MPa) and PA images at 100% laser power ($\text{max fluence}=20\;[{\mathrm{mJ}/\mathrm{cm}}^{2}]$) at an excitation wavelength of 700 nm. About 25 frames of US and PA images were acquired and were subsequently analyzed using MATLAB.

About 10 wt%, 10 kPa polyacrylamide phantoms containing six 1-mm diameter vessels were prepared the day of imaging using degassed, DDI water. The DDI water was degassed using a SRDS-1000 (FUS Instruments, Toronto) water degassing system. For each phantom, the polyacrylamide solution was prepared and poured into a 2  cm×2  cm holder with six parallel fire-polished borosilicate vessels (1-mm O.D). The borosilicate vessels were carefully removed after polymerization leaving 6 hollow channels and the phantom was removed from the holder for temporary storage. In storage, phantoms were hydrated in PBS in a beaker at room temperature.

**Fig. 4:**
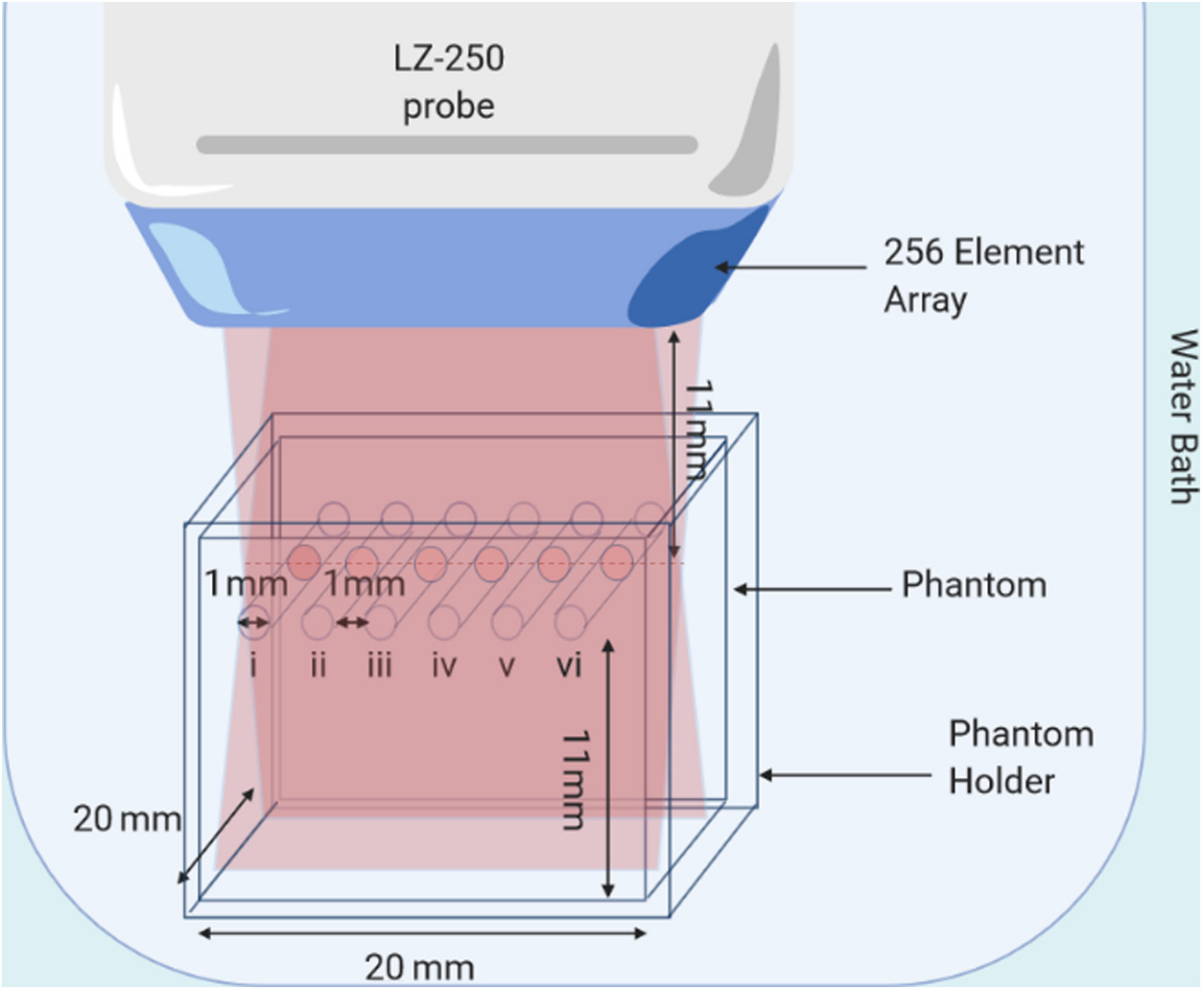
Submerged multimodal US/PA experimental setup; a 10% polyacrylamide phantom with 6 hollow channels (1-mm diam; 1-mm separation) housed in a clear holder to block vessel ends; Vevo LAZR 2100 system with 256-element linear array LZ250 transducer (21 MHz central frequency; 13 to 24 MHz bandwidth) and common US/PA focus at 11-mm imaging depth. Figure created with BioRender.com.

Phantom channels were filled with activated solutions of SBNBs each at a 1 in 30 dilution. The phantom was replaced in the phantom holder and the channels were covered at the ends using glass slides to retain the contents. The sealed phantom-in-holder was placed in a DDI water bath for imaging. The centers of the vessels were approximately aligned at the laser-transducer focus (schematic in Fig. 4). Each phantom was imaged at three different cross sections along the channel length. This process was repeated three times, yielding a total of nine cross sections.

US/PA data from the six contrast agents were analyzed using MATLAB R2019a. The laser energy was recorded for each frame and used to normalize the PA RF-data before further analysis. To analyze the intensity differences for each vessel, a mask was created of circles with centers corresponding to the centers of the vessels and diameters matching vessel diameter (as determined from the US image). The mask was multiplied by the log-compressed RF-matrix to isolate the signal from each vessel. The average signal from a vessel was determined by taking the mean signal within a single vessel region. This was then averaged for all 25 frames and all nine cross sections of the same vessel type. The standard deviation of the mean vessel signal of the nine cross sections was determined.

## 4 Results

### 4.1 Physical Property Characterization

**Fig. 5:**
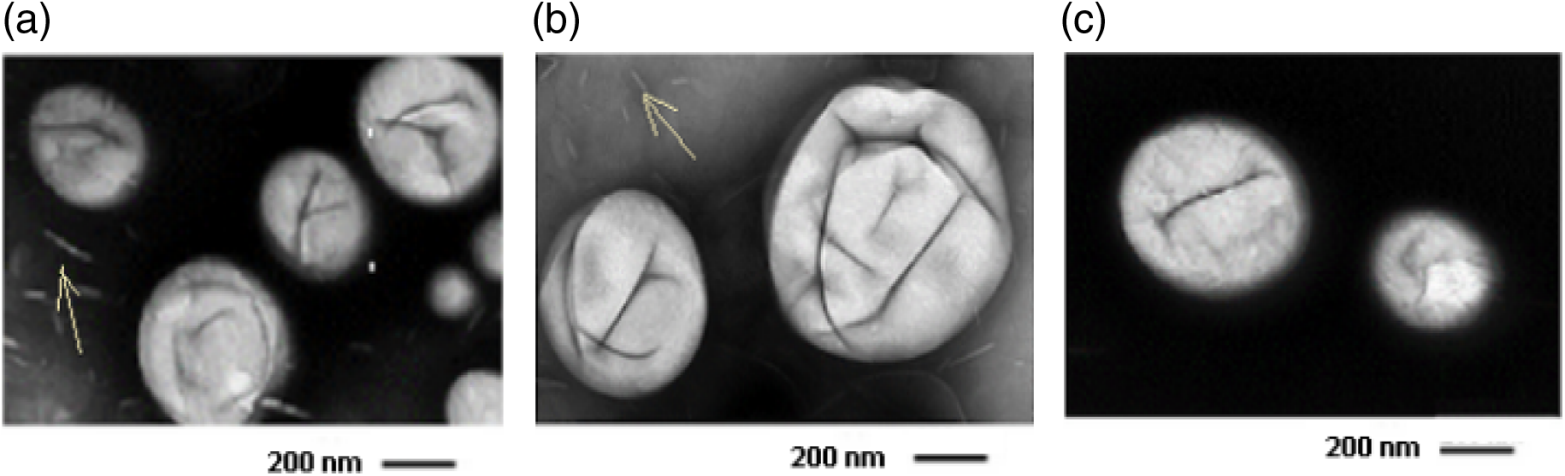
Representative transmission electron microscopy images for lipid-shelled NBs with differing SB B content (a) 0 mg; (b) 0.3; and (c) 0.5  mg/ml in the lipid shell solution.

TEM images of 0 [Fig. 5(a)], 0.3 mg [Fig. 5(b)], and 0.5 mg [Fig. 5(c)]. The dark ridges on the bubble surface may be shell buckling which is thought to play an important role in bubble dynamics.^40^ Also present in the images are flecks (indicated by arrows) which are attributed to nonbuoyant lipid particulates.

**Fig. 6:**
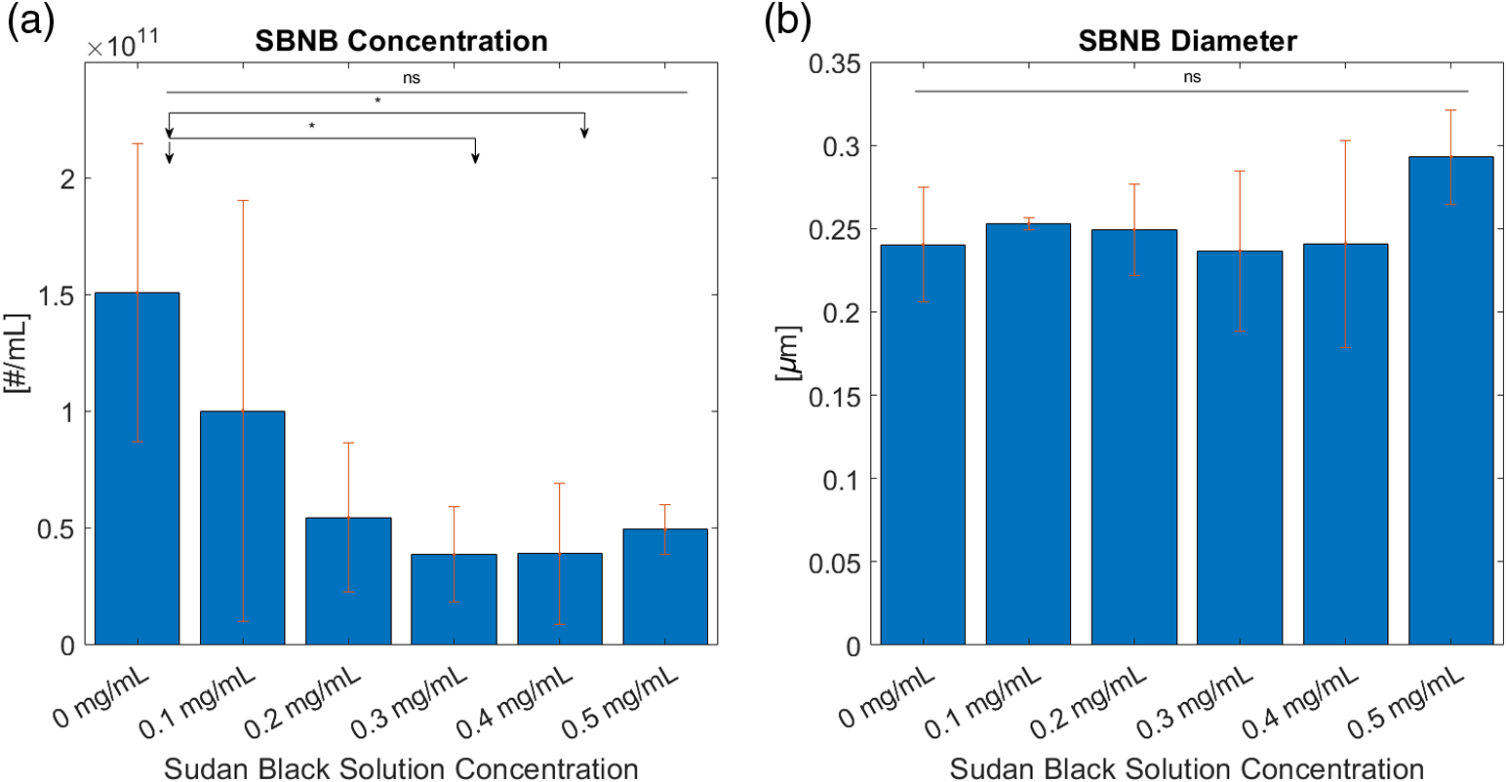
(a) SBNB concentration and (b) diameter from RMM (n=3) differences between the average bubble diameter for varying bubble formulations were also determined to be not significant.

The average buoyant particle concentration and diameter of three measurements are shown for each activated SBNB formulation in Figs. 6(a) and 6(b). From a one-way ANOVA test differences between bubble yields for various SBNB formulations were determined to be not significant (ns). However, from two-tailed t-tests comparing 0.3  mg/ml and 0.4  mg/ml SB to control the concentration was reduced with statistical significance. Differences between the average bubble diameter for varying bubble formulations were determined to be not significant. Overall, we produced high agent yield ($∼10^{11}$) and consistent agent size (range: ∼120 to 700 nm; mean ∼200 to 300 nm).

Successful formulation of safe, stable and, requires the repeatable preparation of homogenous (monodisperse) populations.^46^ The term polydispersity index (PDI) is used to describe the degree of non-uniformity of a size distribution of particles. The numerical value of PDI ranges from 0.0 (a perfectly uniform sample) to 1 (a highly polydisperse sample). This statistic is provided upon measurement from the RMM system and compared here. The PDI was averaged for all samples (See Fig. S4 in the Supplementary Material). The results show the addition of SB B die to the stabilizing lipid shell does not significantly alter the PDI of the population, although there is a slight increasing trend. This was statistically validated by a one-way analysis of variance test (n=3).

### 4.2 Surface Tension

**Fig. 7:**
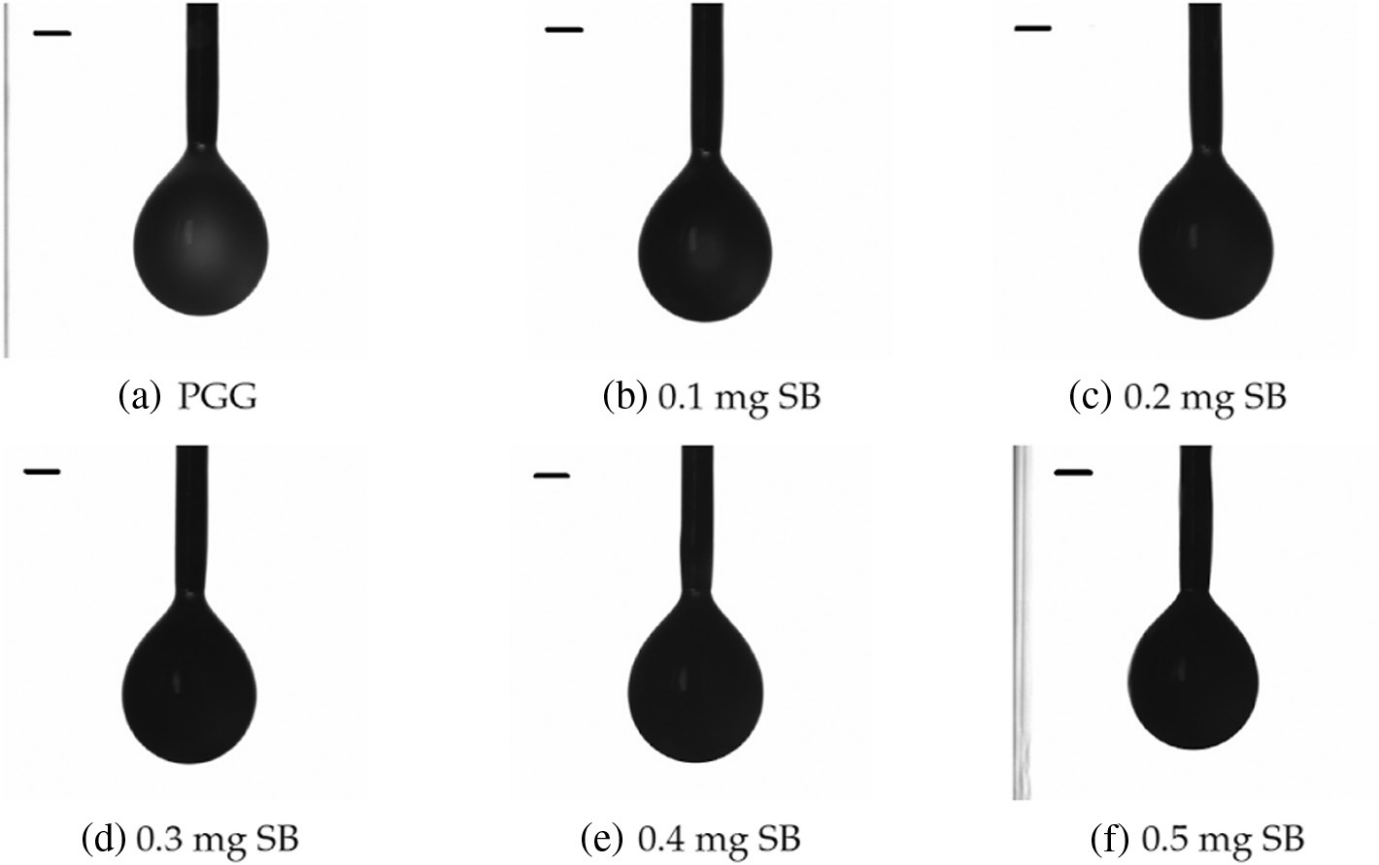
Raw data for pendant drop tensiometry. Profiles of pendant drops of (a) PGG solution and PGG solution with (b) 0.1; (c) 0.2; (d) 0.3; (e) 0.4; (f) 0.5  mg/ml added SB B, respectively, for lipid-shell stabilized NBs; (scale bar=0.5251  mm).

**Fig. 8:**
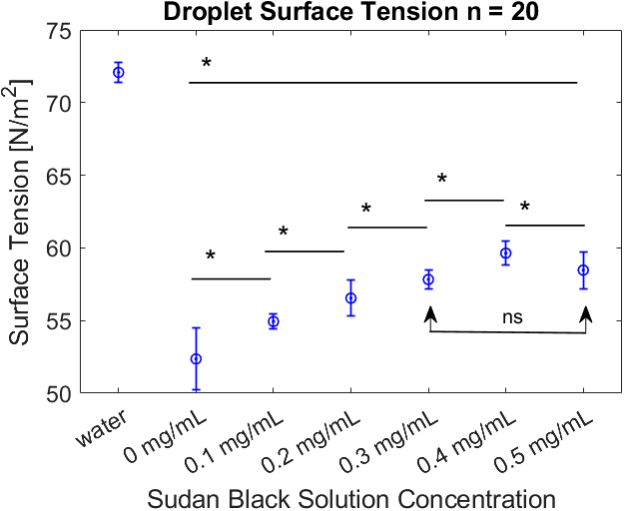
Surface tension as measured from bulk droplets of lipid solution containing varying contributions of SB B dissolved in the lipid. (n=20; bars represent standard deviation).

Samples of the raw data from the pendant drop tensiometry measurements are shown in Fig. 7. The results of the surface tension measurements determined by pendant drop tensiometry are shown in Fig. 8. A multiple comparison test of the mean surface tension of the droplets was performed to identify groups of significance. As shown by the horizontal bars in Fig. 8, all lipid formulations with SB dye were determined to be statistically different (*) from the 0 mg SB formulation. Each formulation type had statistical significance when compared to the formulation with 0.1-mg difference in SB solution concentration. However, the difference between the 0.3-mg lipid and 0.5-mg lipid was not significant (ns). Water droplets were not included in the significance tests. Compared to water droplets, the lipid solutions have a reduced surface tension.

### 4.3 Ultrasound Stability

**Fig. 9:**
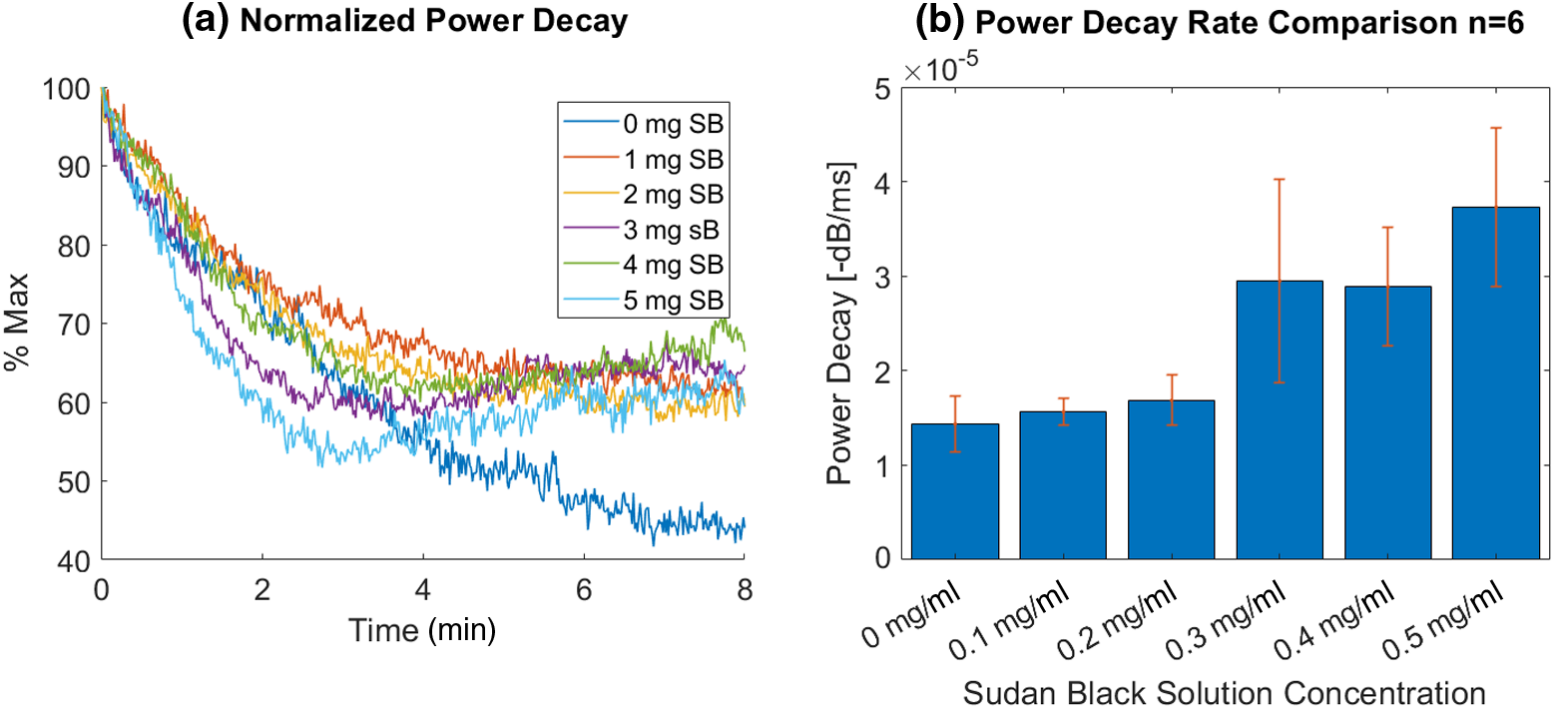
(a) US power decay normalized to maximum power of time series (Toshiba, Tochigi-Ken, Japan, 6-MHz transmit, 12 MHz receive, 240 kPa peak negative pressure, M.I, 0.1) for 1 in 100 dilution NBs in PBS with differing concentrations of SB B in the lipid shell. (b) Bubble decay rates after 8 min continuous US stimulation for lipid-stabilized with varying concentrations of SB B dye in the lipid shell.

Power decay curves from 8 minutes of constant US stimulation are shown in Fig. 9. These power decay curves show a decrease in %-maximum US power with respect to time until 2 min is reached. After the 2-min time point, the 0.3, 0.4, and 0.5 mg SBNB power curves increase. It is possible that this increase is a result of bubble growth and bubble joining. If only the first 2 min of the decay curve are considered, an exponential fit can be used to determine the rate of the population decay in that time window. Exponential fits to un-averaged curves were determined to obtain decay rates for the first 2 min. These decay rates were obtained for all six measurements and subsequently averaged (Fig. 9). With increasing SB contribution an increase in power decay rate is expressed with a sharp increase detected between the 0.2 and 0.3  mg/ml SBNBs and the maximum decay rate detected from the 0.5  mg/ml SBNBs.

### 4.4 Single-Bubble Excitations

**Fig. 10:**
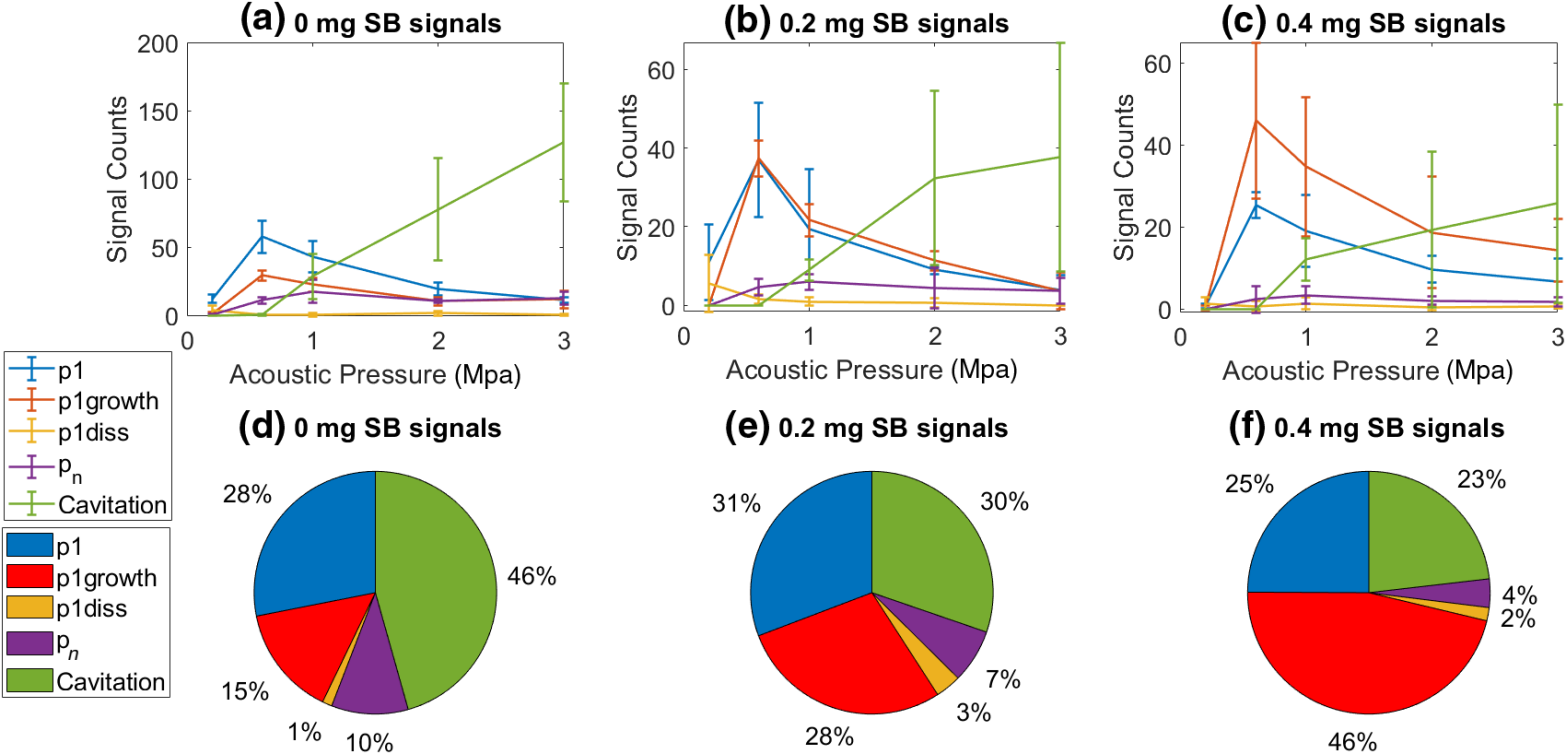
(a)–(c) Single bubble signal count as a function of acoustic pressure. (n=3) and SB contribution in lipid-shell. (d)–(f) Average contribution of signal types to total signal count summed over all pressures (n=3); average total number of signals = 511 (0  mg/ml), 261 (0.2  mg/ml), and 246 (0.4  mg/ml).

The total counts of recorded signals above threshold from a single bubble with respect to acoustic pressure were recorded [Figs. 10(a)–10(c)]. The linear and nonlinear signal counts were compared. To identify the types of signals that most significantly contributed to the total number of signals detected by a particular formulation, the average percent of signals above threshold for each signal type was plotted and compared [Figs. 10(d)–10(f)].

The lowest bubble activity was recorded at the minimum pressure used in the experiments (0.2 MPa) for all three formulations. Here, we use bubble activity to refer to the number of counts registered above threshold. At this lower pressure, the 0-mg SBNB formulation generated the most counts. The bubble behavior by all formulations at 0.2 MPa was dominated by p1 signals. No nonlinear signals were detected at 0.2 MPa for any of the NB formulations. As pressure of the incident acoustic wave increased, an increase in the number of linear and nonlinear counts was recorded.

At 0.6 MPa, the number of ${p1}_{\text{growth}}$ signals increased, reaching a maximum at 0.6 MPa for all NB formulations, and decreased with increasing pressure beyond 0.6 MPa. Additionally, as the SB concentration increased, the number of ${p1}_{\text{growth}}$ signals detected increased (red line) and dominated the number of signals detected. For this pressure, more p1 signals (blue line) were recorded on average than ${p1}_{\text{growth}}$ signals (red line) for the 0  mg/ml NBs. In the case of the 0.2  mg/ml formulation, p1 and ${p1}_{\text{growth}}$ signal counts were approximately equal. For the 0.4  mg/ml formulation, however, the counts of ${p1}_{\text{growth}}$ signals doubled p1 counts.

Beyond 0.6 MPa, the counts of linear signals (p1, p1growth, and p1diss) decreased. A peak in nonlinear signals occurred at 1 MPa for all NB formulations (purple line). A steady increase in bubble cavitation started at 1 MPa and peaked at 3 MPa (green line). No bubble cavitation events were detected before 1 MPa for any of the NB formulations. Due to the low number of signals registered above the threshold that were nonlinear for all NB types, all nonlinear signals were grouped together. Differences in the different types of nonlinear oscillations (p2 and p3) may have gone undetected due to the low overall detection of nonlinear signals.

From average contribution of signal types to total signal count [Figs. 10(d)–10(f)] no apparent trend as a function of SB dye concentration is observed for p1 signals (blue wedge). However, upon the addition of SB dye, the relative number of ${p1}_{\text{growth}}$ signals increases (red wedge). On average, the ${p1}_{\text{growth}}$ signals contribute approximately twice as much to the total signal count of 0.2  mg/ml SBNBs compared to the NBs without dye. Similarly, the ${p1}_{\text{growth}}$ signals contributed three times more to the total 0.4  mg/ml SBNBs than the 0  mg/ml NBs. No apparent trend with increasing dye concentration is observed for ${p1}_{\text{diss}}$ oscillations (yellow wedge).

The number of the various $p_{n}$ signals were low (see Supplementary Material). However, the contribution of all $p_{n}$ signals to the total number of signals detected from the NB formulation decreased with increasing SB concentration tending toward statistical significance (p<0.1) (purple wedge). Additionally, the total contribution of cavitation signals decreased as a function of SB concentration (green wedge). Cavitation signal counts and total signal counts varied greatly across samples and therefore this trend was not established with statistical significance.

### 4.5 Multimodal Imaging

**Fig. 11:**
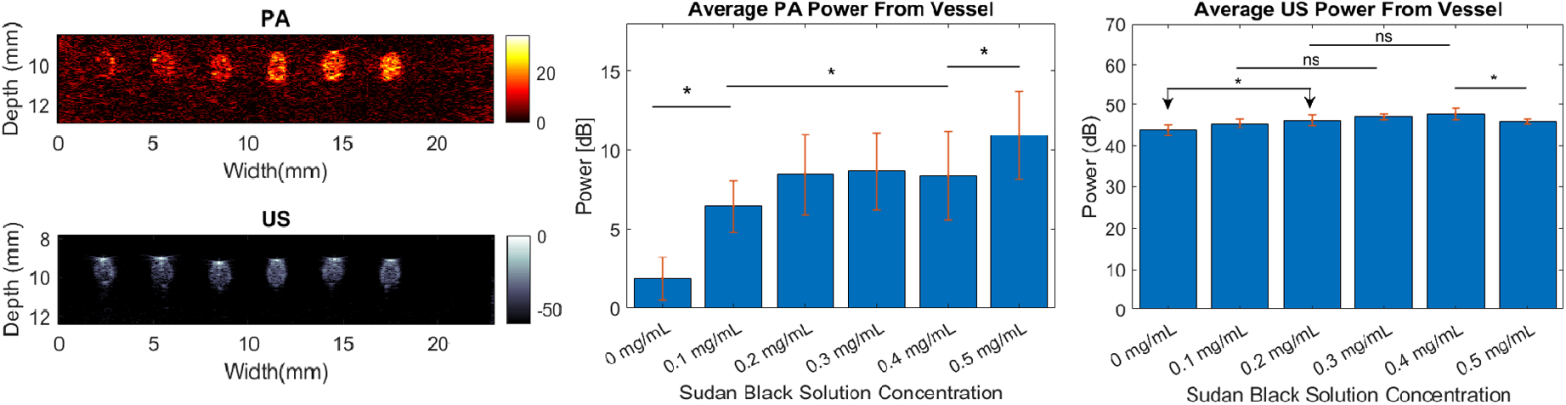
Representative PA(top)/US (bottom) images with channels containing 1:29 dilution of 0 mg, 0.1 mg, 0.2 mg, 0.3 mg, 0.4 mg, 0.5 mg Sudan Black from left to right; average PA (center; n=9) and US (right; n=9) signal from SBNBs.

Multimodal US/PA images were collected using the Vevo LAZR system. A representative image of the six-channel filled SBNBs-PBS dilutions, in order of increasing SB concentration from left to right, is shown in Fig. 11. The top and bottom images are from PA and US imaging, respectively, collected from the same cross section of the phantom channel.

In the PA image, six vessel cross sections are identifiable with increasing PA signal amplitude from left to right. The increase in signal corresponds to the increase in dye contribution. The first vessel (left-most), containing undyed NBs generated little detectable signal due to the low absorption profile of lipids/PFC. The five right-most channels containing SB are most clearly delineated. In the US image, by comparison, all six channels are clearly delineated. High amplitude signal at the top of each vessel cross section in the US images corresponds to differences in acoustic impedance at the phantom-PBS interface at the channel walls. Differences in US backscatter between the bubble populations in the channels were minimal (Fig. 11–bottom left).

An analysis of the average PA and US power is summarized in Fig. 11 (center and right). Horizontal lines marked by a star (*) are used to indicate groups that are statistically different from one another, as determined from one-way ANOVA and multiple comparisons tests. The computed average PA power from the vessel cross sections (Fig. 11–center) shows that an increase in average PA power occurs with increasing SB solution concentration. The average PA power of the undyed bubble formulation was lower compared to all dyed NBs. Additionally, the average PA power of the 0.5-mg bubble formulation was determined to be different from the other NB formulations. The groups with SB dye ranging from 0.1 to 0.4 mg SB integration were determined not to be statistically different from one another.

For the concentration of 0.1-mg/ml SB, there was a 4 dB increase in power in comparison to the control (0  mg/ml). The SBNBs with the highest contrast (0.5  mg/ml) was on average 8-dB higher than the control. Undyed agents produced low PA signals. For dyed SBNBs the PA signal increase with SB concentration was not linear. A plateau in signal is seen in average PA signal plots. The computed average US power from the vessel cross sections (Fig. 11- right) showed a trend of increasing power with increasing SB. A test of significance indicated a statistical difference between 0.1, 0.2, and 0.3  mg/ml SBNBs in a pairwise comparison with undyed NBs. Statistical difference between the average US power of 0.5 mg SBNBs and 0.4-mg SBNBs was also established. However, the groups with SB dye ranging from 0.1 to 0.4 mg SB integration did not show statistically significant differences.

## 5 Discussion

In this work, we examined the effects of SB dye inclusion on the physical and acoustic properties of shell stabilized NBs. Inclusion of a strong optical absorber such as SB can add PA capabilities to acoustic contrast agents, making them suitable for dual-modality imaging. No significant change in morphology was observed with increasing loading of SB (Fig. 5). Additionally, bubble yields were consistently orders of magnitude higher than previously developed agents (our agents $∼10^{11}\text{ bubbles}/\mathrm{ml}$ as compared to $∼10^{9}\text{ bubbles}/\mathrm{ml}$ from other groups aiming to produce PA/US agents^3^^,^^47^^–^^49^). In general, the addition of SB to the NBs resulted in lower bubble concentration. Notably, a 40%+ reduction in bubble yield was measured for the bubbles made with 0.3 and 0.4  mg/ml SB compared to undyed bubble formulations.

Despite the consistent morphology of the agents seen in TEM and the consistent size measured using RMM, a steady increase in surface tension was measured as a function of SB contribution, peaking at0.4  mg/ml. The maximum change in surface tension measured was from the 0.4  mg/ml lipid formulation (13.9% higher than the control). Beyond 0.4 mg, the measured difference in surface tension decreased such that the surface tension of the 0.5 and 0.3 mg SB solutions were approximately equal. The 0.3-mg/ml samples consistently produced the lowest bubble concentration (Fig. 6), and corresponded with a plateau in surface tension, and a sudden increase in bubble instability (Fig. 9). This trend could be an indication of a critical concentration of SB in the lipid shell solution between 0.3 and 0.4  mg/ml wherein the shell is fully saturated by SB. Further addition of SB beyond this concentration may not lead to SB incorporated into the bubble membrane, and therefore reduces the impact on the bubble material properties. A future study of SB precipitation may address the question.

Surface tension values were measured of lipid droplets at the air–lipid interface instead of measurements of lipid bilayers at water–lipid and PFC–lipid interfaces, introducing limitations to the measurements. Because the measurements were collected for droplets, the surface tension measurements presented here are not an accurate representation of the interfaces of a NB and are only used to identify trends. Additionally, because the SBNBs feature a PFC gas core, a more accurate measurement of the NB tension would use PFC. However, evidence has been presented to suggest that the decrease in surface tension for the PFC–lipid interface in comparison to the air–lipid interface is approximately linear.^50^ Therefore, the differences in surface tension observed here is assumed to exhibit the same trend for the PFC–lipid interface of a lipid droplet. Overall, SB increases the surface tension at the air–lipid interface.

Surface tension differences might be expected to have an impact on stability under US stimulation. Recently, changes in membrane surface tension at different degrees of compression via optical tensiometry experiments were shown to impact bubble stability.^50^ Similarly, the US power decay from prolonged US stimulation measured here suggests that an increase in SB B integration in lipid shell material destabilizes the SBNB formulations. The observed increase in surface tension as a result of SB addition shown in Fig. 8 is consistent with the observed instability of a bulk population of SBNBs [Fig. 9(b)] under prolonged US stimulation.

As NB solutions are subject to prolonged US stimulation (Fig. 9), bubble cavitation occurs. The number of bubbles remaining depends on the initial bubble concentration and the rate of cavitation events. As the bubble population decays due to cavitation events, the measured US power should decrease exponentially. However, under the same conditions, bubble fragmentation and/or coalescence can occur. Acting as competing processes to bubble NB cavitation, scattering from larger bubbles formed during coalescence would cause deviations from an exponential decay curve. Bubble fragmentation or coalescence events could result in higher US backscatter compared to bubble populations for which significant bubble destruction has occurred and may offer an explanation for the increase in signal with time observed for 0.3-0.5  mg/ml SBNB curves [Fig. 9(a)]. Finally, if the bubble size increases by absorbing gas from the surroundings the US backscatter could also increase. These bubble dynamics offer possible explanations for the increase in US power detected after the 2-minute time point in the US decay curves [Fig. 9(a)] at higher SB concentrations.

As shown in Fig. 10, the addition of SB to the bubble lipids increases the tendency for gas exchange across the NB shell, and therefore increases the shell permeability. This was observed by an increase in bubble growth behavior at 0.6 MPa with increasing SB. Our single-bubble excitation findings of shell permeability are consistent with the findings of Sarkar et al.^51^ In their work, added interfacial elasticity resulted in different dissolution dynamics when interpreted through an analytical expression for the dissolution time for an encapsulated air bubble.^51^ They showed that increasing the permeability of the shell linearly decreases the dissolution time, and therefore increases the rate of gas exchange across the shell. Dissolution of the SBNBs under Laplace pressure drives the lipid molecules to pack into a tensionless state.^51^ It is energetically favorable for shells with higher surface tension to undergo gas exchange across the shell. Gas exchange dynamics can be linked to the growth and dissolution signals for 0.3−0.5  mg/ml SBNBs (with the highest measured surface tensions Fig. 8). Calculating and comparing the slopes of the dissolution signals could be an area of future study for measuring comparable shell permeability with new surfactants. Leong et al. also showed that at high surface loading of a surfactant the bubble growth rate was significantly dependent upon the type and charge of the headgroup.^52^ This offers another avenue for future investigations.

Due to the increased Laplace pressure for NBs, the effects of increased shell permeability and rates of dissolution would be more prevalent for NB populations compared to MB populations. The disproportionate count of growth signals in comparison to ${p1}_{\text{diss}}$ signals measured in SBNB experiments [Figs. 10(d)–10(f)] may be interpreted in several ways. In the case of a PFC-core bubble investigated by Sarkar et al.,^51^ a bubble with initial radius of 1 mm would dissolve in 2500 s, much longer than the 30-cycle time frame investigated here (1.4 ms). The integration of SB might increase the thickness of the SBNB shell and consequently increase the thickness of the liquid-air mass transfer boundary. What is referred to as the “shell effect” describes the tendency for larger boundary layer thickness to promote more gas to enter the bubble during expansion than out during the compression phase.^53^ This is also known as rectified diffusion. Moreover, the surface area of the NBs can increase as a function of SB concentration without an increase in NB volume if shell buckling also increases. Shell buckling is the term used to describe folding of the shell into the core such that the bubble is not perfectly spherical and such that compression leads to an unstable situation where the monolayer buckles out of plane.^54^ If the SB forces a separation of the lipid surfactants, the surface area of the shell would also increase. According to the “area effect,” more gas tends to enter the bubble during bubble expansion when the surface area is larger.^53^

We also observed a decrease in total nonlinear signal counts [Figs. 10(d)–10(f)] which supports the hypothesis that the presence of SB decreases nonlinear behavior of the NBs. One possible explanation of the observed decrease in nonlinear signals could be that SB increases the elasticity of the bubble shell. This would occur if the SB molecules forced a separation of the lipid molecules, as described above. A more robust investigation on the effect of SB on NB nonlinear oscillations and shell buckling requires in-depth analysis and is an area of future work.

Periodic patterns in RF lines from a single bubble response are an indication of harmonic nonlinear behavior of the agent response to the driving US wave. The capacity for gas-core lipid vesicles to significantly enhance US contrast is largely attributed to their high degree of nonlinearity. Specialized pulse sequences have been developed to capitalize on this nonlinear behavior which allow for the complete suppression of complex tissue signal (see pulse inversion imaging,^55^ amplitude modulation contrast enhanced imaging^56^ and contrast harmonic imaging^57^).There has also been increasing interest in the use of US contrast agents for drug and gene delivery.^58^ The identification of enhanced bubble dissolution or gas exchange is critical in the context of agents adapted for drug delivery. Increased leakage of the contexts from a drug-loaded bubble core would result in delivery to undesired or untargeted locations. Furthermore, increased tendency for bubble destruction, also termed inertial cavitation, could be beneficial for focused US therapies in which the high-power emission from a bubble burst can cause enhanced damage by thermal deposition on undesired tissue such as cancer.^58^^,^^59^ Alternatively, highly stable agents may be more desirable for longevity, vascular tracking, and molecular imaging.^58^ Recognizing the periodic and non-periodic patterns in the time domain allows the user to select the most adequate agent for their purposes.

In comparison to single bubbles (Fig. 10), the average US signal from bulk SBNBs was detected in parallel with PA measurements, which exhibited an increasing power with SB contribution (Fig. 11). The differences between average US power detected from the various SBNBs [Fig. 11(b)] may be a consequence of differences in bubble concentration or bubble fragmentation and/or coalescence kinetics. The PA imaging results support the use of SB to increase the absorptive properties of lipid shelled NBs. Low contrast enhancement from the low-absorbing bubble population is possible and was also observed. The lipid shelled bubbles can locally increase optical scattering.^60^ By increasing optical scattering, the local fluence increases and the likelihood of optical absorption increases. This can translate to a stronger PA signal. From PA data an increase of signal with increased SB was observed, which reached a plateau at 0.3 mg SB. SB has an absorption spectrum that is dependent on its molecular environment.^27^ If the SB absorption spectra shift as a result of the decreased presence of lipid molecules or increased presence of other SB molecules, the absorption at 700 nm may decrease. Alternatively, if a critical SB integration threshold occurs between 0.3 and 0.4  mg/ml, as is suggested by surface tension measurements, the increase in signal beyond 0.4 mg may be caused by unbound or precipitous SB. This threshold is common among all measurements in this study. Precipitation of SB can be confirmed by testing further contributions of SB beyond 0.5  mg/ml for sedimentation of the dye. One final explanation for the observed plateau in PA contrast enhancement could be US attenuation. Although there is no evidence of SB-dependent attenuation in the PA images, it is possible that differences in acoustic attenuation caused by changed shell properties of the SBNBs are affecting the PA signals. Comparing the data with images of an equal volume of nonbuoyant particles can determine the effects of the gas core on PA amplitude of SBNBs.

## 6 Conclusions

We successfully produced SBNBs with consistently high efficiency ($∼10^{11}\;\mathrm{NBs}/\mathrm{ml}$), important for contrast enhancement in clinical settings. We also showed that integration of the SB dye agent modifies the shell properties of US contrast agents by increasing lipid surface tension at the lipid-gas interface. Differences in surface tension correlated with decreased bubble stability, as shown from prolonged SBNB exposure to US excitation. This knowledge is of value in particular because the use of nanoscale agents for imaging a tumor, where enhanced permeability and retention effects would allow accumulation of the NBs, require longevity. From the single-bubble excitations, we established that the integration of SB dye into the lipid-shell of a NB increases the rectified diffusion of NBs in response to US stimulation. For a population of SBNBs under US excitation, the presence of SB produced minimal differences in US signals compared to undyed bubble populations. We also showed simultaneous enhancement in contrast for both US and PA imaging, where PA enhancement was SB concentration dependent and US enhancement was not.

In terms of molecular imaging, both PA and US imaging typically suffer from strong background signals. The dual-modality PA/US imaging system with a combination of the dual-modality agent, SBNBs, can be applied to image cancers, monitor drug delivery, and visualize internal organs such as bladders and lymph nodes without being restricted to the vasculature due to the size of the agents. The increase in rectified diffusion is attributed to physical changes to the shell through increase in surface tension and enhanced shell permeability. The presence of additional components in a NB shell has an effect on the US scattering from the NB. Understanding how NBs behave when changes are made to shell composition is required for the optimal use of NB formulations in US and PA multimodal imaging. From a clinical point of view, NBs are increasingly being considered for clinical practices. The safety profile of SB dye has not been sufficiently studied for immediate translation. Clinically feasible imaging scanners have been utilized in this study to showcase the potential of such an agent.
