# Supplementary material for: Effects of shell-integrated Sudan Black dye on the acoustic activity and ultrasound imaging properties of lipid-shelled nanoscale ultrasound contrast agents
Source: J Biomed Opt. 2022 Jan 21;27(1):016501. doi: 10.1117/1.JBO.27.1.016501 (PMC8781525; doi:10.1117/1.JBO.27.1.016501)
Supplement: Supplementary file 1 [file JBO_027_016501_SD001.docx]

The Effects of Shell-Integrated Dye on the Acoustic Properties of Ultrasound Contrast Agents

Dana Wegierak,^a,c^ Grace Fishbein,^a^ Eric Abenojar,^b^ Al De Leon,^b^ Jinle Zhu,^b^ Yanjie Wang,^a^ Charlotte Ferworn,^a^ Agata A. Exner,^b,c,*^ Michael C. Kolios^,a,*^

aRyerson University, Faculty of Science , Department of Physics, Toronto, Canada

bCase Western Reserve University, Department of Radiology, Cleveland, United States

cCase Western Reserve University, Department of Biomedical Engineering, Cleveland, United States

# Supplementary Material

The following data are provided to offer the reader supporting information to the methods presented.

Table S1. Lipid Sample Contents and amounts for preparation of Sudan Black Dyed Nanobubbles; DBPC, DPPA and DPPE obtained from Corden Pharma (Switzerland), and DSPE-mPEG obtained from Laysan Lipids (Arab, AL).

| **Material** | **Amount** |
| --- | --- |
| (DBPC) 1,2-dibehenoyl-sn-glycero-3-phosphocholine | 60.1 mg |
| (DPPA) 1,2-dipalmitoylsn-  glycero-3-phosphate | 10 mg |
| (DPPE) 1,2-dipalmitoyl-sn-glycero-3-phosphoethanolamine | 20 mg |
| (DSPE-mPEG) 1,2-distearoyl-sn-glycero-3-phosphoethanolamine-N  -[methoxy(poly(ethylene glycol))-2000] | 10 mg |
| Propylene Glycol | 1.03 g |
| Phosphate Buffered Saline | 8 mL |
| Glycerol | 1 mL |


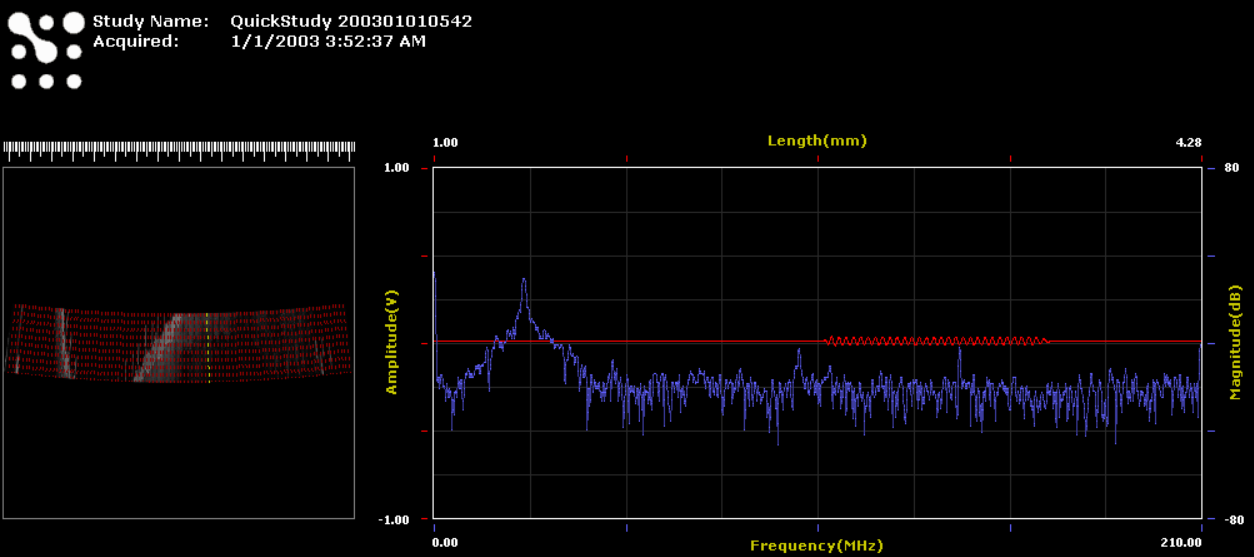


**Fig. S1** Representative system data of a 0 mg PGG NB sample stimulated by a 30 cycle pulse train at 32 MHz; time (red) and frequency (blue) representations of the selected line.


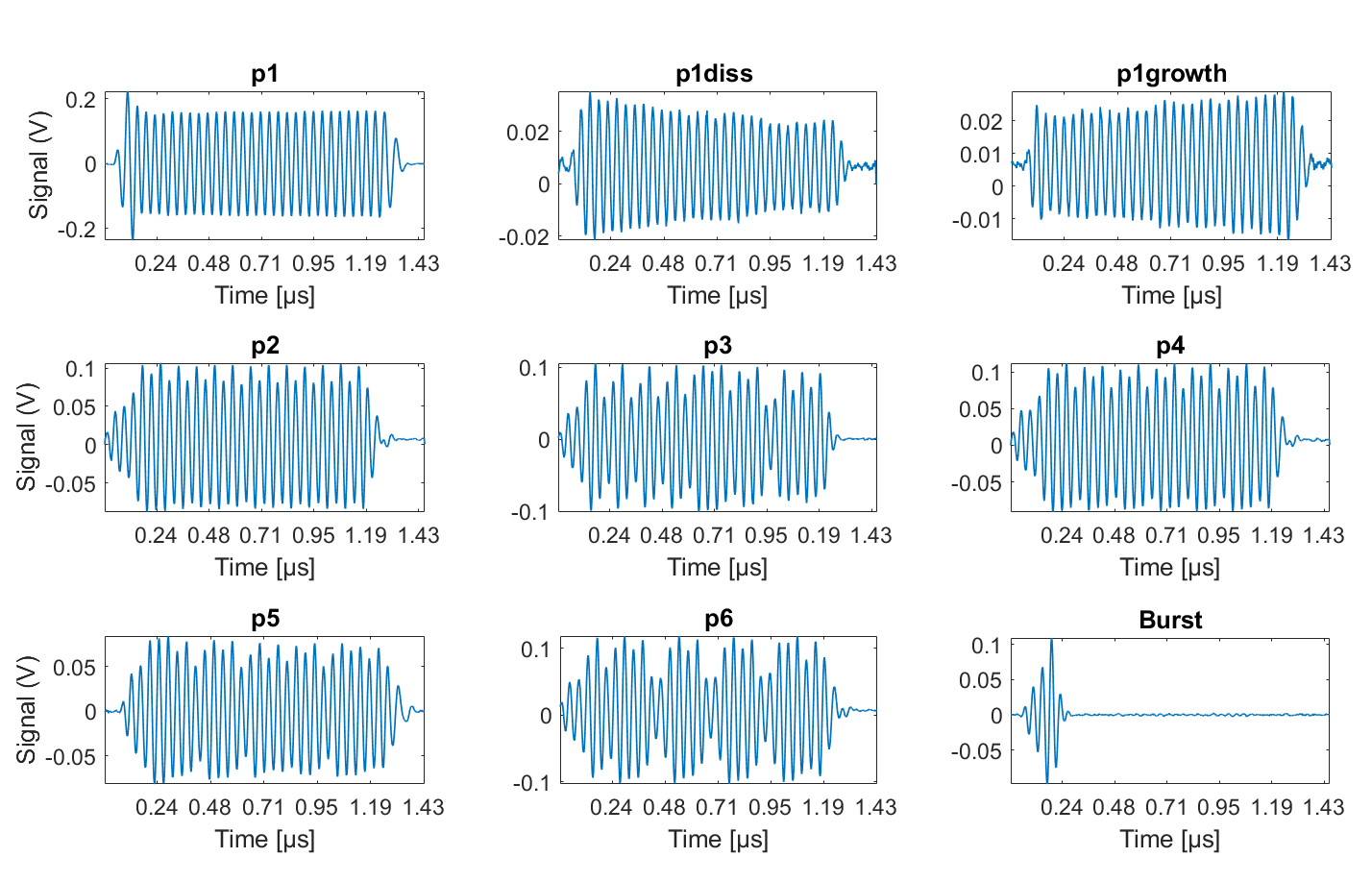


**Fig. S2** Representative RF-lines of linear and nonlinear signals from single nanobubbles stimulated for 30 cycles at 25 MHz central frequency

**
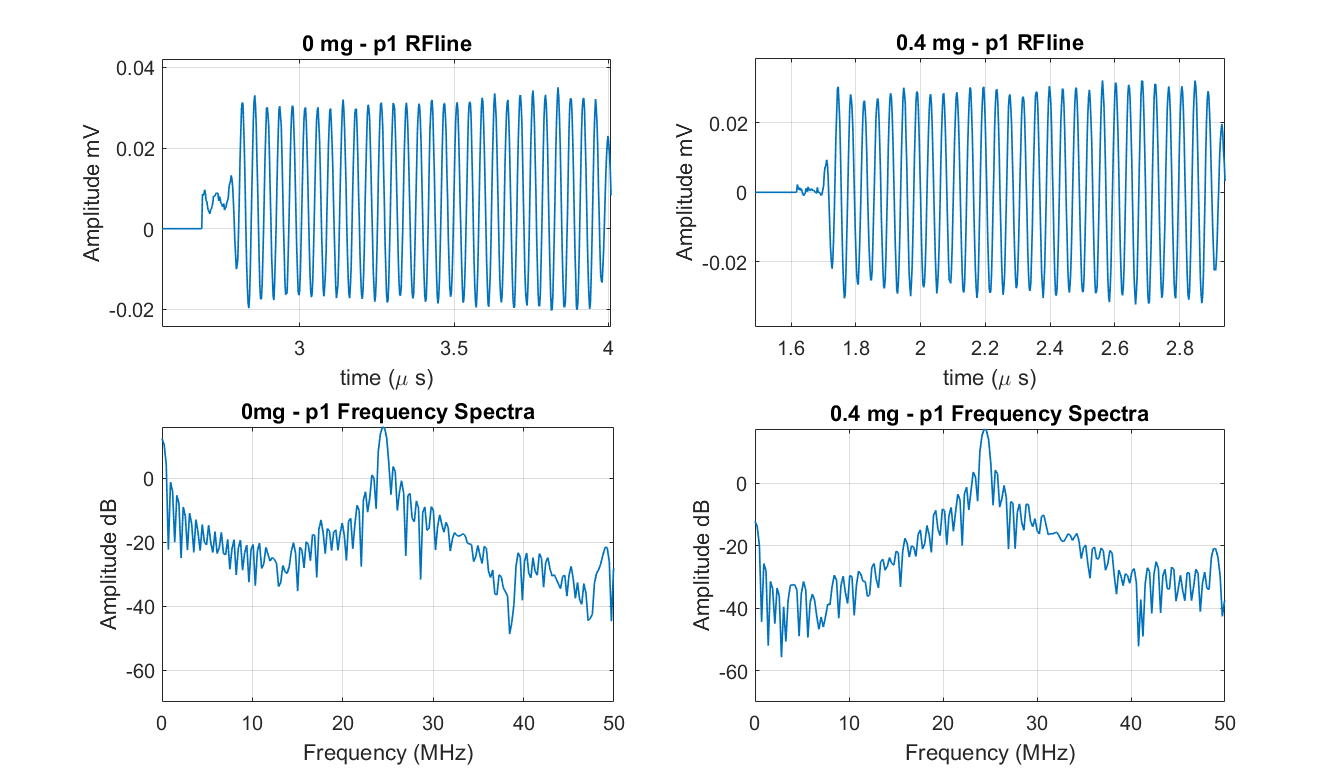
**

**Fig. S3** RF-lines (top row) and Frequency Spectra (bottom) from single nanobubbles with 0 mg/mL (left) and 0.4 mg/mL (right) Sudan Black B stimulated for 30 cycles at 25 MHz central frequency and 0.6 MPa incident pressure

**
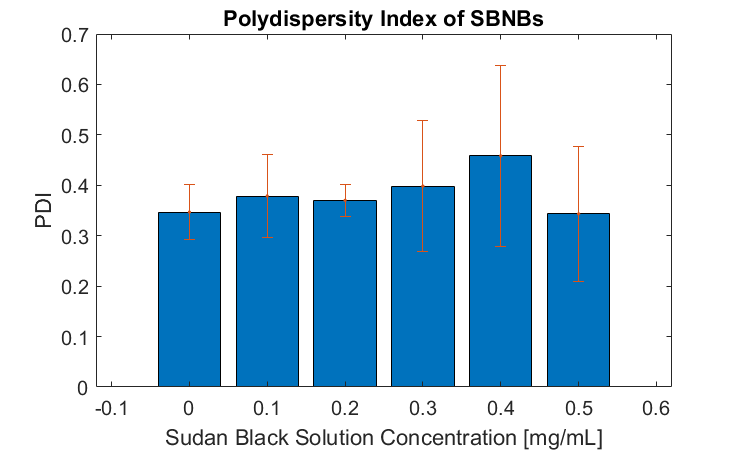
**

**Fig. S4** Polydispersity Index (PDI) of Sundan Black B dyed nanobubbbles with increasing concentration of Sudan Black B integrated into the lipid shell. Data shown represents mean PDI +/- standard deviation between three separate formulation batches (n=3).
